# Early Indicators of Cognitive Decline in Online Financially Active Older Adults

**DOI:** 10.1093/geroni/igaa057.3304

**Published:** 2020-12-16

**Authors:** Kimberly Grzesek Nora Mattek, Nicole Sharma, Katherine Wild

**Affiliations:** Oregon Health & Science University, Portland, Oregon, United States

## Abstract

As access to computers and use of technology becomes more common in older adults, the incidence of online financial fraud increases. Law enforcement officials and fraud experts predict this trend to continue as aging baby boomers increasingly become targets. One reason this population might be at risk for financial fraud is due to subtle, undetected decline in cognitive abilities that have been associated with decline in financial capacity. Many cases of incipient cognitive decline go undetected. Further, those with early cognitive decline often have poor insight to its potential impact on daily functioning. Assessment of Activities of Daily Living Skills (ADLs) is paramount to determine early decline in daily activities. The ORCATECH Life Lab was designed to evaluate subtle neurological and other health changes and their relation to changes in daily functioning. Older adults participating in the Life Lab complete annual ADL and neurocognitive assessments. Additionally, 97 participants completed an online technology questionnaire where 64 participants reported participating in online financial activity. Results revealed that within the online financially active group, some assistance in ADL’s was required. However, inconsistencies in ADL change over time highlight the challenges of screening for early signs of mild cognitive impairment (MCI) in patients that fall between normal cognition and MCI. In-home information technology may help overcome these challenges. Defining subtle changes in ADLs is a crucial step to enable early diagnosis of neurocognitive disorders and assist health care providers in improving disease management and to prevent incidents of financial fraud in this vulnerable population.

